# Smoking habit and chemo-radiotherapy and/or surgery affect the sensitivity of *EGFR* plasma test in non-small cell lung cancer

**DOI:** 10.1186/s13104-020-05209-9

**Published:** 2020-08-03

**Authors:** Vinh Thanh Tran, Thang Thanh Phan, Son Truong Nguyen, Bich-Thu Tran, Toan Trong Ho, Suong Phuoc Pho, Tran Bao Nguyen, Tuyen Thi Bich Pham, Anh Tuan Le, Vu Thuong Le, Hang Thuy Nguyen

**Affiliations:** 1 The Laboratory D Unit, Clinical Cancer Center, Cho Ray Hospital, 201B Nguyen Chi Thanh Street, Dist. 5, Ho Chi Minh City, 700000 Vietnam; 2 Faculty of Biology-Biotechnology, University of Science, VNU-HCM, Ho Chi Minh City, 700000 Vietnam; 3 Department of the Vice Minister, Ministry of Health, Hanoi City, 100000 Vietnam; 4 Department of Chemo-Radiotherapy, Clinical Cancer Center, Cho Ray Hospital, Ho Chi Minh City, 700000 Vietnam; 5 Department of Thoracic Disease, Cho Ray Hospital, Ho Chi Minh City, 700000 Vietnam; 6 Department of Clinical Pathology, Cho Ray Hospital, Ho Chi Minh City, 700000 Vietnam

**Keywords:** *EGFR* mutations, cfDNA, NSCLC, Sensitivity

## Abstract

**Objective:**

This study aimed to identify the influential factors for the sensitivity of epidermal growth factor receptor (*EGFR*) plasma test in non-small cell lung cancer (NSCLC). The mutations were detected in tumor tissue and matched plasma samples from 125 newly diagnosed adenocarcinoma, clinical-stage IIIB-IV patients, and compared the diagnostic values of *EGFR* plasma test between groups of clinical characteristics. The influential factors for the sensitivity were identified and assessed by logistic regression.

**Results:**

*EGFR* mutations were detected in 65 (52.0%) tumor tissue and 50 (40.0%) matched plasma samples (*P *= 0.028). Compared to the tissue method, the concordance rate, sensitivity, and specificity of the *EGFR* plasma test were 86.4%, 75.4%, and 98.3%, respectively. Notably, we found that sensitivity of the test is higher in non-smokers (84.1%) compared to smokers (57.1%, *P *= 0.018), and in treatment naïve subjects (85.7%) compared to whom undergone chemo-radiotherapy with/without surgery before testing (56.5%, *P *= 0.009). Furthermore, the highest sensitivity was attained in patients without these two factors (90.3%), whilst the lowest value was noted in those with both factors (40.0%, *P *= 0.004). The multivariable analysis confirmed that smoking habit and treatment history have independently negative impacts on sensitivity (OR = 0.24, *P *= 0.019, and OR = 0.36, *P *= 0.047, respectively).

## Introduction

After the breakthrough of using EGFR tyrosine kinase inhibitors in treatment for NSCLC, *EGFR* mutation testing in the non-invasive samples as peripheral blood has opened a new period of precision medicine. More than thirty studies have been conducted in the past 15 years to assess the diagnostic values and applicability of this test in clinical practice [1–6]. Most of them have shown that the specificity of the *EGFR* blood test is quite high (95–98%) [1]. However, the agreement of results in plasma with the tumor tissue is widely-fluctuated (66–100%) [1–6]. Also, the sensitivity of the *EGFR* plasma test is very different between laboratories and areas (17–100%) [1–6]. Some factors have been shown to influence the sensitivity and accuracy of the test like method of circulating free DNA (cfDNA) extraction and mutation detecting, sample type, sample volume, and processing method [1, 7–9]. We assume that other clinical factors also affect the concordance rate and sensitivity of the test. In the laboratory setting, we use a high-throughput system together with a recommended protocol to isolate cfDNA [7, 8], and scorpion amplification-refractory mutation system (scorpion ARMS) to detect *EGFR* mutations, and compare the diagnostic values of the test between groups of clinical characteristics.

## Main text

### Materials and methods

#### Patients and samples

A total of 125 newly diagnosed adenocarcinoma, clinical-stage IIIB-IV NSCLC patients were selected for this study at Cho Ray hospital from Jan-2016 to August-2019 (approval number 212-BVCR-HDDD). Patients were asked for participation and signed consent forms before collecting samples for *EGFR* mutation analysis (5 mL of peripheral blood and matched tumor tissues after confirming histology assessments). Among them, blood samples of 69 patients were collected on the same day of performing tumor biopsy procedures. Whereas in 29 other cases, blood samples were obtained after 1–4 cycles of chemo-radiotherapy (1–7 weeks after diagnosis). In 27 patients who had undergone tumor resection, blood samples were collected within 2 weeks after surgery. No one has been treated with targeted therapies like EGFR tyrosine kinase inhibitors before the mutation testing. The clinical information such as Ecog PS (Cooperative oncology group performance status), smoking habit, clinical-stage, organ metastasis, and tumor size was selected from the medical records.

#### Genomic DNA extraction from tumor tissue and EGFR analysis

The genomic DNA (gDNA) was extracted from 3 sections of formalin-fixed paraffin-embedded (FFPE) tumor tissue samples using QIAamp DNA FFPE Tissue kit (Cat No./ID: 56404) according to the instruction of the manufacturer (Qiagen, Hilden, Germany). Concentration and purity of gDNA were measured by NanoDrop 8000 Spectrophotometer (Thermo Scientific, USA) and adjusted to 2 ng/μl before sequencing. *EGFR* mutations (exon 19 deletion–*EGFR*^E19del^, a threonine-to-methionine point mutation in exon 20–*EGFR*^T790M^, a leucine-to-arginine point mutation in exon 21–*EGFR*^L858R^) were detected by pyrosequencing method using Therascreen EGFR Pyro kit (Cat No./ID: 971480) according to the instructions of the manufacturer (Qiagen, Hilden, Germany).

#### Circulating free DNA extraction from plasma and EGFR analysis

The blood samples were prepared and used for cfDNA extraction on the QIAsymphony machine, as described in previous work [10]. *EGFR* mutations in cfDNA were detected by the scorpion ARMS method, the Therascreen EGFR Plasma RGQ PCR kit (Cat No./ID: 870311), performed on the RotorGene Q 5Plex HRM platform according to the manufacturer’s instructions (Qiagen, Hilden, Germany).

#### Statistical analysis

Fisher’s exact test was used to compare the relative frequencies between groups. The concordance rate between the *EGFR* plasma and the *EGFR* tumor tests was determined by the Kappa statistic [11]. The mutation results in the tumor tissue were used as the standard reference for calculating sensitivity, specificity, positive and negative predictive values of *EGFR* plasma test. Variables with *P* value  ≤ 0.25 in sensitivity comparing were selected to use in the multivariable analysis by logistic regression. The influential factors for the sensitivity were identified and assessed by the odds ratio (OR) with a 95% confidence interval (95%CI). All data analyses were done by R statistical software v.3.5.1 (R foundation, 1020 Vienna, Austria). *P *< 0.05 was considered statistically significant.

### Results

#### Baseline characteristics

All of 125 cases enrolled in this study were newly-diagnosed, clinical-stage IIIB-IV adenocarcinoma with a median age of 59 (95%CI 57–61) years. On general examination, 24 cases (19.2%) were scored ≥ 2 with the Ecog PS criteria. All of the female patients (37 subjects) were never smokers, while 71 of 88 (80.7%) male patients were smokers or ever smokers.

The diagnostic imaging results just before plasma-mutation testing indicated that 83 (66.4%) patients have the lymph-node metastasis while 39 (31.2%) cases have the pleural effusion. Lung metastasis and distant metastasis were observed in 39 (31.2%) and 100 (80.0%) cases, respectively. Of which, brain, bone, liver, and other metastasis (including stomach, adrenal, spleen, thyroid gland, and prostate metastases) were observed in 36 (28.8%), 37 (29.6%), 27 (21.6%), and 49 (39.2%) cases, respectively. Eighty-two (65.6%) cases have tumor size > 5 cm.

#### EGFR status in tumor tissue and plasma samples

*EGFR* mutations were detected in tumor tissue and plasma of 65 (52.0%) and 50 (40.0%) cases, respectively (*P *= 0.028, Additional file 1: Table S1). Patients who have undergone chemo-radiotherapy and/or tumor removal have the lower frequency of *EGFR* mutations in tumor tissue (41.0%) and plasma (25.0%) compared to others (60.8%, *P *= 0.028; and 52.2%, *P *= 0.002, respectively, Additional file 2: Table S2). Besides, in both tumor tissue and plasma samples, *EGFR* mutations occurred with a higher rate in non-smokers and female patients compared to the remaining groups (*P *< 0.001).

#### The diagnostic values of EGFR plasma test and influential factors for sensitivity

Among 125 patients, 108 cases have the same results in plasma and matched tumor tissue samples resulting in a pooled concordance rate of 86.4% between methods (Additional file 3: Table S3). The sensitivity and specificity of the plasma test reached 75.4% and 98.3%, respectively. Among the clinical sub-groups, the concordance rate and the sensitivity of *EGFR* plasma test were higher in patients with Ecog PS ≥ 2, pleural effusion, and tumor size > 5 cm compared to others but not with statistical significance (P > 0.05, Table 1). Notably, we found that sensitivity of *EGFR* plasma test is higher in non-smokers (84.1%) compared to smokers (57.1%, *P *= 0.018), and higher in untreated patients (85.7%) compared to others (56.5%, *P *= 0.009, Table 1). Furthermore, the highest sensitivity was archived in non-smoking untreated patients (90.3%) while the lowest value was observed in smoking treated subjects (40.0%, *P *= 0.004). In the multivariable analysis, smoking habit (OR = 0.24, *P *= 0.019) and treatment history as chemo-radiotherapy and/or surgery (OR = 0.36, *P *= 0.047) were identified as independent factors for the lower sensitivity of *EGFR* plasma test (Table 2).

**Table 1:** The diagnostic values of *EGFR* plasma test between groups

Variable	Concordance		Sensitivity		Specificity	
	Percentage (%)	*P*-value	Percentage (%)	*P*-value	Percentage (%)	*P*-value
Mutation type						
*EGFR*^E19del^ (n = 125)	92.8	0.212	77.5	0.472	100.0	0.360
*EGFR*^L858R^ (n = 125)	95.2		78.2		99.0	
Age						
≥ 59 (n = 57)	87.7	0.694	79.3	0.510	96.4	0.467
< 59 (n = 68)	85.3		72.2		100.0	
Gender						
Female (n = 37)	83.8	0.290	80.0	0.424	100.0	0.714
Male (n = 88)	87.5		71.4		98.1	
Ecog PS						
0–1 (n = 101)	84.2	0.191	70.6	0.159	98.0	0.833
≥ 2 (n = 24)	95.8		92.9		100.0	
Smoking status						
No (n = 54)	87.0	0.428	84.1	*0.018*	100.0	0.652
Yes (n = 71)	85.9		57.1		98.0	
Clinical stage						
IIIB (n = 6)	83.3	0.592	66.7	0.578	100.0	0.950
IV (n = 119)	86.6		75.8		98.3	
Lung metastasis						
No (n = 86)	84.9	0.334	73.3	0.404	97.6	0.683
Yes (n = 39)	89.7		80.0		100.0	
Lymph-node						
No (n = 42)	85.7	0.874	75.0	0.956	100.0	0.700
Yes (n = 83)	86.8		75.6		97.6	
Pleural effusion						
No (n = 86)	83.7	0.155	70.5	0.152	97.6	0.700
Yes (n = 39)	92.3		85.7		100.0	
Distant metastasis						
No (n = 25)	88.0	0.545	80.0	0.461	100.0	0.833
Yes (n = 100)	86.0		74.0		98.0	
Tumor size						
≤ 5 cm (n = 43)	84.2	0.233	70.0	0.164	97.0	0.700
> 5 cm (n = 82)	90.7		84.0		100.0	
Treatment: chemo-radiotherapy and/or surgery						
No (n = 69)	91.3	0.076	85.7	*0.009*	100.0	0.362
Yes (n = 56)	80.4		56.5		97.0	
Treatment/Smoking status						
No/No (n = 34)	91.2	0.418	90.3	*0.004*	100.0	0.483
No/Yes or Yes/No (n = 55)	87.3		70.8		100.0	
Yes/Yes (n = 36)	80.6		40.0		96.2	

Significant values are shown in italic

**Table 2:** Influential factors for the sensitivity in multivariable analysis

Variable	OR (95%CI)	*P*-value
Ecog PS: ≥ 2 versus 0–1	4.81 (0.59–46.80)	0.176
Smoking habit: Yes versus No	0.24 (0.07–0.79)	*0.019*
Pleural effusion: Yes versus No	1.09 (0.26–4.56)	0.911
Tumor size: > 5 cm versus ≤ 5 cm	1.79 (0.51–6.32)	0.366
Treatment history: Yes versus No	0.36 (0.10–0.91)	*0.047*

In the logistic regression analysis, patients with *EGFR* mutations in both tumor tissue and plasma were coded 1, while the positive cases only in the tumor tissue were coded 0; other factors were coded 1: Ecog PS ≥ 2, smoking habit = yes, pleural effusion = yes, tumor size > 5 cm, and treatment history = yes; the remaining groups of factors were coded 0

Significant values are shown in italic

### Discussion

Previous studies have shown that the concordance rate and sensitivity of *EGFR* plasma test are different between centers depend on the technique used and pre-analytic factors [1, 7, 9]. The deep-sequencing, digital polymerase chain reaction (dPCR), and scorpion ARMS are the best methods in detecting mutations in cfDNA. Of these technologies, digital PCR and scorpion ARMS are widely-used up to date because of the low limit of detection (0.05–0.1%), convenience, rapidity, and low cost [12].

In this study, we used the scorpion ARMS method to detect *EGFR* mutations in plasma samples and showed a high agreement with the tumor tissues together with a moderate sensitivity and excellent specificity (Additional file 3: Table S3). The limited sensitivity (75%) is due to nearly 25% of the cases with false-negative results in the plasma. It is worth noting that *EGFR* mutations in plasma are detected by the scorpion ARMS method that has a lower limit of detection than the pyrosequencing method (≈ 5%) [13]. We performed a literature review and noted that the false-negative phenomenon also is observed in other studies used scorpion ARMS method [14–28], dPCR [5, 6, 29], and even ultra-deep sequencing methods [2–4, 30]. Therefore, the *EGFR* plasma sensitivity might be affected by the other factors besides the technique influences.

**Fig. 1:**
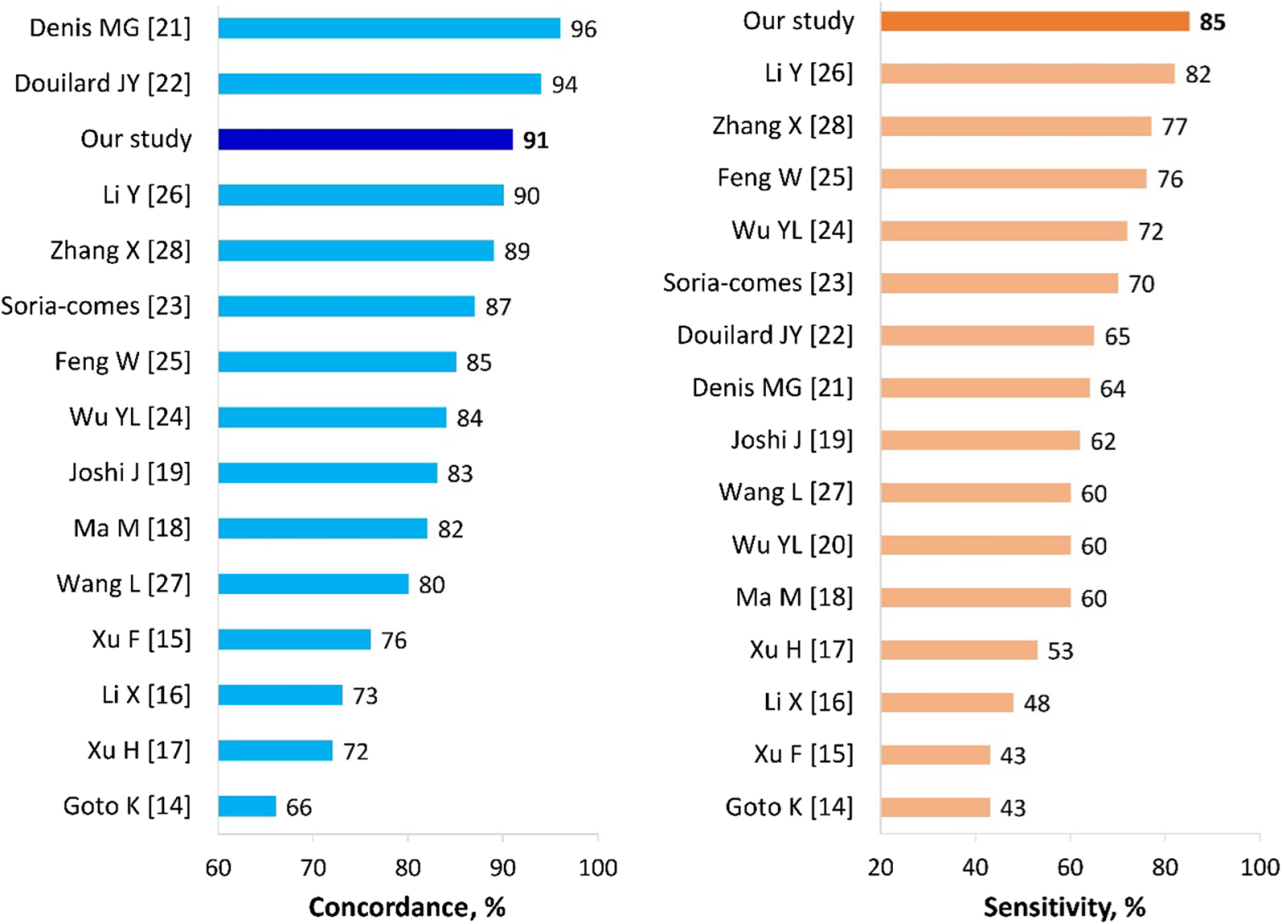
The concordance rate and sensitivity of *EGFR* plasma test in the studies used scorpion ARMS method

Our data indicated that the concordance rate and sensitivity of *EGFR* plasma test are improved in untreated patients (Table 1), and higher than values in previous studies used the same method (Fig. 1). It is easy to see that these studies used the column-based method in cfDNA extraction [14–28] that lead to the low-yield of cfDNA and low sensitivity [9]. In the study, we also found that smoking habits influence the presence of *EGFR* mutations in plasma (Additional file 2: Table S2) and thus affect the sensitivity of the test (Table 1). The combination of this factor with induction therapy as chemo-radiotherapy and/or tumor removal before mutation testing resulted in very low sensitivity, whereas the highest value was archived in those without these two factors (Table 1). Finally, the independent negative impact of these factors on sensitivity was confirmed by the multivariable analysis (Table 2).

The negative impact of treatment history on *EGFR* plasma sensitivity can be explained by the reduction of mutant-cell derived cfDNA fraction. Guo N reported that 92% of mutation frequencies decrease within two days after surgery [31]. Likewise, a deep clearance of *EGFR* mutant alleles after chemotherapy was showed in the study of Mok T [32]. Taniguchi K and Bai H demonstrated that both *EGFR*-mutated and non-mutated cell clones coexist in the same tumor [33, 34]. Because of more sensitivity to chemotherapy, the *EGFR*-mutated cancer cells are eliminated faster than non-mutated cells leading to the shift of *EGFR* status from positive to negative after chemotherapy [33, 34]. Zhang S noted that chemotherapy is an independent factor for the presence of *EGFR* mutations in the plasma of NSCLC patients [35].

The association of smoking with the low *EGFR* frequency in tumor tissue and plasma samples of NSCLC has been shown in many studies [16, 18, 20, 36]. Nevertheless, the underlying mechanism of this phenomenon is unclear to date. Previous studies have shown that smoking is related to an elevated tumor mutational burden (TMB) in NSCLC [37–41]. Moreover, the higher TMB is associated with tumor protein p53 (*TP53*) alterations, and negatively associated with *EGFR* mutations [41]. Other studies emphasized the association of smoking habit with the presence of *TP53* and Kirsten rat sarcoma viral oncogene (*KRAS*) mutations rather than *EGFR* mutations [42–44]. We assume that in smoking NSCLC patients, *EGFR* mutation is not the key driver, and the *EGFR* mutant subclone might present at a low fraction in the tumor content. Therefore, the amount of tumor DNA released by these cells into the blood is limited (it might be below 0.05% of the total cfDNA amount). That is the cause of the low sensitivity of plasma testing.

In clinical practice, these results help the clinicians to define subgroups of patients with different sensitivity of *EGFR* plasma test. In those confirmed with lung cancer by the diagnostic imaging and protein tumor markers but failure with the biopsy procedure, or inadequate tumor tissue for *EGFR* mutation analysis, clinical doctors should require the mutation testing in plasma before starting chemo-radiotherapy. In other cases, mutation analysis in the resected tumors should be done first for the patients who have undergone surgery.

### Conclusion

The results of this study showed that the sensitivity of the *EGFR* plasma test is lower in smoking treated patients compared to others. Besides, smoking habit and induction therapy as chemo-radiotherapy and/or surgery might be the independently influential factors for the sensitivity.

## Limitations

This study highlights the negative impact of smoking habit and chemo-radiotherapy and/or surgery on the sensitivity of the *EGFR* plasma test in NSCLC. However, this is just an observation from a single-center study. Besides, the sample size in the study is still limited. Further research on a large cohort should be done to confirm this finding. In those studies, one same technique should be used for both tumor and plasma testing to clarify the association of clinical factors with sensitivity.

## Supplementary information

**Additional file 1: Table S1.** Frequency of *EGFR* mutations in tumor tissue and plasma.

**Additional file 2: Table S2.***EGFR* mutation status between groups of patient’s characteristics.

**Additional file 3: Table S3.** The diagnostic values of *EGFR* plasma test.

## Data Availability

The datasets generated during and/or analysed during the current study are available from the corresponding author on reasonable request.

## Abbreviations

ARMS: Amplification-refractory mutation system
cfDNA: Circulating free DNA
dPCR: Digital polymerase chain reaction
EGFR: Epidermal growth factor receptor
Ecog PS: Cooperative oncology group performance status
FFPE: Formalin-fixed paraffin-embedded
gDNA: Genomic DNA
KRAS: Kirsten rat sarcoma viral oncogene
NSCLC: Non-small cell lung cancer
OR: Odds ratio
TMB: Tumor mutational burden
TP53: Tumor protein p53
95%CI: 95% Confidence interval
