# Supplementary material for: Smoking habit and chemo-radiotherapy and/or surgery affect the sensitivity of EGFR plasma test in non-small cell lung cancer
Source: BMC Res Notes. 2020 Aug 3;13:367. doi: 10.1186/s13104-020-05209-9 (PMC7398354; doi:10.1186/s13104-020-05209-9)
Supplement: Supplementary file 1 — Additional file 1: Table S1. Frequency of EGFR mutations in tumor tissue and plasma. [file 13104_2020_5209_MOESM1_ESM.docx]

**Table S1.** Frequency of *EGFR* mutations in tumor tissue and plasma

| ***EGFR* status** | | **In tumor tissue (n=125)** | **In plasma (n=125)** |
| --- | --- | --- | --- |
| Negative, n (%) | | 60 (48.0) | 75 (60.0) |
| Positive, n (%) | | 65 (52.0) | 50 (40.0) |
|  | *EGFR*^E19del^, n (%) | 39 (31.2) | 31 (24.8) |
|  | *EGFR*^E19del+T790M^ | 1 (0.8) | 0 (0.0) |
|  | *EGFR*^L858R^, n (%) | 22 (17.6) | 18 (14.4) |
|  | *EGFR*^L858R+T790M^ | 1 (0.8) | 1 (0.8) |
|  | *EGFR*^T790M^, n (%) | 2 (1.6) | 0 (0.0) |
| *P*-value | | **0.028** | |
