# Supplementary material for: Smoking habit and chemo-radiotherapy and/or surgery affect the sensitivity of EGFR plasma test in non-small cell lung cancer
Source: BMC Res Notes. 2020 Aug 3;13:367. doi: 10.1186/s13104-020-05209-9 (PMC7398354; doi:10.1186/s13104-020-05209-9)
Supplement: Supplementary file 2 — Additional file 2: Table S2.EGFR mutation status between groups of patient’s characteristics. [file 13104_2020_5209_MOESM2_ESM.docx]

**Table S2.** *EGFR* mutation status between groups of patient’s characteristics

| **Variable** | | ***EGFR* status in tumor tissue**  **(n=125)** | | |  | ***EGFR* status in plasma**  **(n=125)** | | |
| --- | --- | --- | --- | --- | --- | --- | --- | --- |
|  |  | (-) | (+) | *P*-value |  | (-) | (+) | *P*-value |
| Age, years (median: 59; 95%CI: 57-61) | | | | | | | | |
|  | ≥59 | 28 | 29 | 0.818 |  | 33 | 24 | 0.660 |
|  | <59 | 32 | 36 |  |  | 42 | 26 |  |
| Gender | | | | | | | | |
|  | Female | 7 | 30 | **<0.001** |  | 13 | 24 | **<0.001** |
|  | Male | 53 | 35 |  |  | 62 | 26 |  |
| Ecog PS | | | | | | | | |
|  | 0–1 | 50 | 51 | 0.490 |  | 64 | 37 | 0.115 |
|  | ≥2 | 10 | 14 |  |  | 11 | 13 |  |
| Smoking status | | | | | | | | |
|  | No | 10 | 44 | **<0.001** |  | 17 | 37 | **<0.001** |
|  | Yes | 50 | 21 |  |  | 58 | 13 |  |
| Clinical stage | | | | | | | | |
|  | IIIB | 3 | 3 | 0.621 |  | 4 | 2 | 0.544 |
|  | IV | 57 | 62 |  |  | 71 | 48 |  |
| Lung metastasis | | | | | | | | |
|  | No | 41 | 45 | 0.914 |  | 52 | 34 | 0.875 |
|  | Yes | 19 | 20 |  |  | 23 | 16 |  |
| Lymph-node | | | | | | | | |
|  | No | 18 | 24 | 0.413 |  | 24 | 18 | 0.643 |
|  | Yes | 42 | 41 |  |  | 51 | 32 |  |
| Pleural effusion | | | | | | | | |
|  | No | 42 | 44 | 0.781 |  | 54 | 32 | 0.344 |
|  | Yes | 18 | 21 |  |  | 21 | 18 |  |
| Brain metastasis | | | | | | | | |
|  | No | 45 | 44 | 0.367 |  | 52 | 37 | 0.572 |
|  | Yes | 15 | 21 |  |  | 23 | 13 |  |
| Bone metastasis | | | | | | | | |
|  | No | 43 | 45 | 0.766 |  | 55 | 33 | 0.379 |
|  | Yes | 17 | 20 |  |  | 20 | 17 |  |
| Liver metastasis | | | | | | | | |
|  | No | 44 | 54 | 0.186 |  | 56 | 42 | 0.214 |
|  | Yes | 16 | 11 |  |  | 19 | 8 |  |
| Other metastasis | | | | | | | | |
|  | No | 33 | 43 | 0.202 |  | 47 | 29 | 0.601 |
|  | Yes | 27 | 22 |  |  | 28 | 21 |  |
| Tumor size | | | | | | | | |
|  | ≤5cm | 18 | 25 | 0.320 |  | 22 | 21 | 0.144 |
|  | >5cm | 42 | 40 |  |  | 53 | 29 |  |
| Treatment: chemo-radiotherapy and/or surgery | | | | | | | | |
|  | No | 27 | 42 | **0.028** |  | 33 | 36 | **0.002** |
|  | Yes | 33 | 23 |  |  | 42 | 14 |  |
