# Supplementary material for: Smoking habit and chemo-radiotherapy and/or surgery affect the sensitivity of EGFR plasma test in non-small cell lung cancer
Source: BMC Res Notes. 2020 Aug 3;13:367. doi: 10.1186/s13104-020-05209-9 (PMC7398354; doi:10.1186/s13104-020-05209-9)
Supplement: Supplementary file 3 — Additional file 3: Table S3. The diagnostic values of EGFR plasma test. [file 13104_2020_5209_MOESM3_ESM.docx]

**Table S3.** The diagnostic values of *EGFR* plasma test

| ***EGFR* status** | | **In tumor tissue** | | |
| --- | --- | --- | --- | --- |
|  |  | (-) | (+) | Total |
| **In plasma** | (-) | 59 | 16 | 75 |
|  | (+) | 1 | 49 | 50 |
|  | Total | 60 | 65 | 125 |
| Kappa | | 0.73 (*P*<0.001) | | |
| Concordance, % (95%CI) | | 86.4 (80.4–92.4) | | |
| Sensitivity, % (95%CI) | | 75.4 (63.1–85.2) | | |
| Specificity, % (95%CI) | | 98.3 (91.1–100.0) | | |
| Positive predictive value, % (95%CI) | | 98.0 (89.4–100.0) | | |
| Negative predictive value, % (95%CI) | | 78.7 (67.7–87.3) | | |
